# Surface-Engineered Graphene Quantum Dots Incorporated into Polymer Layers for High Performance Organic Photovoltaics

**DOI:** 10.1038/srep14276

**Published:** 2015-09-22

**Authors:** Jung Kyu Kim, Sang Jin Kim, Myung Jin Park, Sukang Bae, Sung-Pyo Cho, Qing Guo Du, Dong Hwan Wang, Jong Hyeok Park, Byung Hee Hong

**Affiliations:** 1 Department of Mechanical Engineering, Stanford University, Stanford, California 94305, USA; 2 Department of Chemistry, College of Natural Sciences, Seoul National University, Gwanak-ro 1, Gwanak-gu, Seoul, 151-747, Republic of Korea; 3 Soft Innovative Materials Research Center, Korea Institute of Science and Technology, Eunha-ri san 101, Bongdong-eup, Wanju-gun, Jeollabukdo, 565-905, Republic of Korea; 4 Institute of High Performance Computing, 1 Fusionopolis Way, #16-16 Connexis North, 138632, Singapore; 5 School of Integrative Engineering, Chung-Ang University, 221 Heukseok-dong, Dongjak-gu, Seoul 156-756, Republic of Korea; 6 Department of Chemical and Biomolecular Engineering, Yonsei University, 50 Yonsei-ro, Seodaemun-gu, Seoul 120-749, Republic of Korea

## Abstract

Graphene quantum dots (GQDs), a newly emerging 0-dimensional graphene based material, have been widely exploited in optoelectronic devices due to their tunable optical and electronic properties depending on their functional groups. Moreover, the dispersibility of GQDs in common solvents depending on hydrophobicity or hydrophilicity can be controlled by chemical functionalization, which is particularly important for homogeneous incorporation into various polymer layers. Here we report that a surface-engineered GQD-incorporated polymer photovoltaic device shows enhanced power conversion efficiency (PCE), where the oxygen-related functionalization of GQDs enabled good dispersity in a PEDOT:PSS hole extraction layer, leading to significantly improved short circuit current density (J_sc_) value. To maximize the PCE of the device, hydrophobic GQDs that are hydrothermally reduced (rGQD) were additionally incorporated in a bulk-heterojunction layer, which is found to promote a synergistic effect with the GQD-incorporated hole extraction layer.

Donor-acceptor blended bulk heterojunction (BHJ) organic photovoltaic devices (OPVs) are considered promising next generation solar cells due to their low-cost, light weight, flexibility, and solution processability^1^,^2^,^3^. However, because the thickness of BHJ layer was restricted, the insufficient carrier mobility of the BHJ have limited its light absorption capability^4^,^5^. Therefore, numerous researches have endeavored to enhance the power conversion efficiency (PCE) of the devices by incorporating methods such as introducing newly designed and synthesized donor or acceptor molecules, embedding metal nanoparticles into organic layers, modifying the bimolecular morphology, or introducing interlayer engineering to the electron or hole extraction layer^6^,^7^,^8^,^9^,^10^,^11^. Especially the light absorption and charge transport performances were considerably ameliorated by incorporating shape-controlled Ag or Au nanomaterials such as nanoparticles or nanorods into the polymer layers in OPVs^12^,^13^,^14^. It is because the OPVs can utilize the incident light scattering and surface plasmon (SPR) effects of the metal nanometarials. Furthermore, the high electric conductance of the embedded metal nanomaterials increase the charge transport performance within the polymer layers^13^,^14^,^15^,^16^,^17^,^18^. However, the metal nanomaterials might inflict damage or a short circuit problem on the devices owing to the undesired morphological distortions in the embedded layers or aggregation of the nanomaterials^19^,^20^. Furthermore, metal nanomaterials have lots of potential to induce monomolecular recombination, acting as trap sites and resulting in an inefficient hole extraction from the BHJ layers^20^,^21^. Recently, insulator coated nanostructures such as SiO_2_ coated Au or Ag nanoparticles have also been embedded in the BHJ or poly(3,4-ethylenedioxythiophene):poly(4-styrenesulfonate) (PEODT:PSS) layers^22^,^23^. Although this process can alleviate the risk of short circuit and improve the PCE, the particle size is out of proportion to the OPVs, because it is difficult to say the 80 nm particles are deeply embedded in the 80 nm thick BHJ layer or 40 nm thick PEDOT:PSS layer^20^. In addition, it is difficult to tune the optoelectronic properties of the nanoparticles owing to the control problem of their size or shape.

Recently, our group introduced the graphene quantum dots (GQDs) in the BHJ layer of OPVs^24^. GQDs have been considered as an emerging material for optoelectronic applications, due to their tunable band gap, low toxicity, environmental compatibility and chemical inertness^24^,^25^,^26^,^27^,^28^,^29^,^30^,^31^,^32^,^33^,^34^,^35^. The optical and electrical properties of GQDs were readily tuned by controlling their chemical functionalities. Noticeable enhancement of PCE was recorded by incorporating reduced GQDs (rGQD) into BHJ layer only in very small quantities. Moreover, the possibility of the short circuit was evitable within the GQD-embedded polymer layers because GQDs exhibit non-metallic behaviors with sufficient band gap energy unlike the conventional metal nanomaterials^24^.

In this study, to maximize the dispersion stability of GQDs in PEDOT:PSS (AI4083), we synthesized GQDs with sufficient oxygen based functionalities by using a simple process based on the acidic treatment of carbon fibers^36^. The GQDs were well dispersed in the polar solvent and PEODT:PSS without any severe aggregations due to the oxygen-related functionalities on the surface of GQDs. In order to investigate their positive effects in OPVs, the BHJ layer composed of thieno[3]-thiophene/benzodithiophene (PTB7) and [6,6]-phenyl C_71_ butyric acid methyl-ester (PC_71_BM) was spin coated on top of the GQDs incorporated PEDOT:PSS layer. The fabricated devices with GQDs showed high efficient polymer based BHJ solar cells without risking the irrecoverable damages. Furthermore, the additionally incorporated rGQD in the BHJ layer were found to promote a synergistic effect with the GQD-incorporated hole extraction layer.

## Results and Discussion

**Figure 1:**
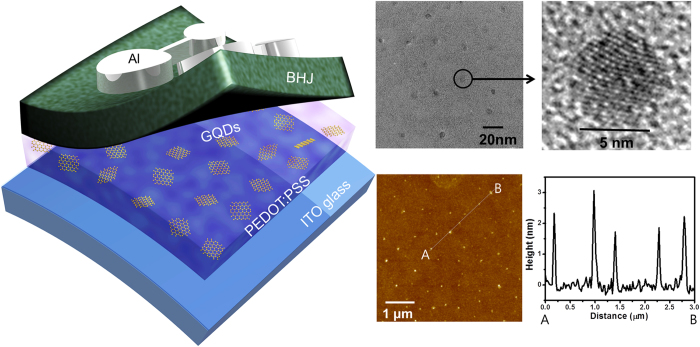
Schematic of device, and TEM and AFM images of GQDs. Schematic of OPV device with a GQD-incorporated PEDOT:PSS layer, and TEM images of the GQDs. The scale bar is 20 nm on the TEM image, and 5 nm on the inset image. AFM image of GQDs (5 μm by 5 μm) and height distribution from A to B.

**Figure 2:**
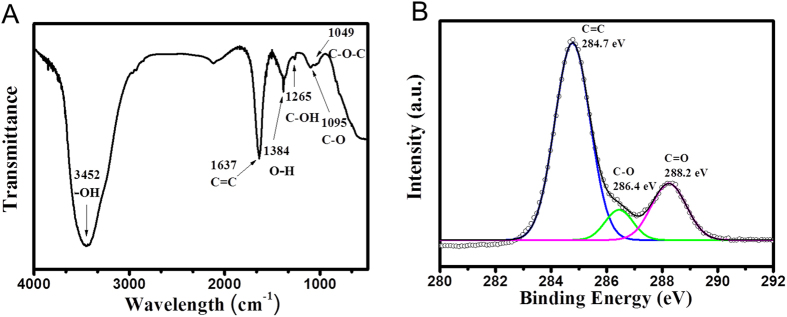
Characteristics of GQDs. FT-IR (**A**) and XPS C1s (**B**) spectra of GQDs.

Figure 1 displays transmission electron microscope (TEM) and atomic force microscope (AFM) images of as-synthesized GQDs with uniform diameter of ~5 nm. The height of GQDs were ~2 nm, which reflect the number of layers in GQDs was approximately 3–4 layers, as evident from the AFM line profile. Fourier Transform Infrared Spectroscopy (FT-IR) and X-ray Photoelectron Spectroscopy (XPS) measurements were carried out to determine the composition of GQDs. Figure 2A shows FT-IR spectrum of GQDs in the range of 4000 ~ 400 cm^−1^, and exhibits the characteristic absorption bands corresponding to the stretching and bending vibration of -OH groups at 3452 cm^−1^, C=C stretching at 1637 cm^−1^, O-H deformation vibration at 1384 cm^−1^, phenolic hydroxyl group stretching of C-OH groups at 1265 cm^−1^, C-O vibration groups at 1095 cm^−1^, and epoxy stretching vibration of C-O-C groups at 1049 cm^−1^. In the C1s XPS spectrum of GQDs in Fig. 2B, three different peaks were decomposed, centered at 284.7, 286.4 and 288.2 eV, corresponding to sp^2^ carbon aromatic rings (C=C), C-O and C=O, respectively. The distinguished C-O and C=O peaks imply that the oxygen-related functional groups such as hydroxyl and carboxyl groups are on the edges of GQDs. Therefore, the functionalities could contribute to the dispersion of GQDs in polar solvents. In order to incorporate the GQDs into the PEODT:PSS solution, as-synthesized GQDs were blended in methanol, and were mixed with the PEDOT:PSS solution. The methanol does not have significantly negative effects on device performances, because it has been widely used as an additive or co-solvent for the PEDOT:PSS layer to improve the film morphology^37^.

### Surface morphologies of the GQDs-incorporated PEDOT:PSS film

**Figure 3:**
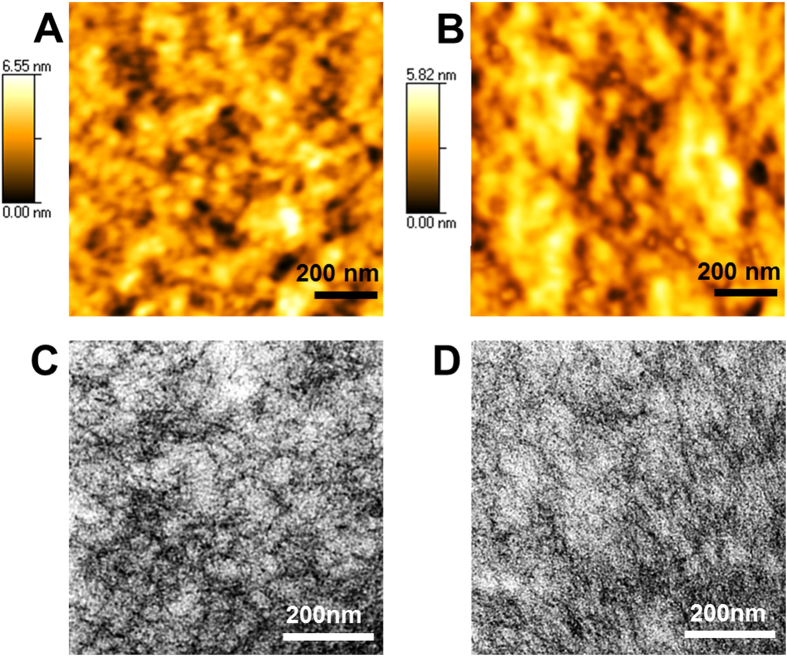
AFM and TEM images of PEDOT:PSS film. AFM images of PEDOT:PSS films without GQDs (**A**) and incorporating GQDs (**B**), which were spin coated on top of the ITO glass. Spherical aberration corrected transmission electron microscopy (Cs-corrected TEM) images of PEDOT:PSS film without (**C**) and with (**D**) GQDs. GQD concentration was 0.4 wt.%

GQD-incorporated PEDOT:PSS layers were prepared by incorporating the GQDs/methanol solution in the PEDOT:PSS solution with varying weight ratio from 0.2 to 0.8 wt.%. Optimum device efficiency of 8.17% was accomplished by incorporating 0.4 wt.% of GQDs into the PEDOT:PSS layer as shown in supplementary Fig. S1 online. Though significantly lower quantity of GQDs were used in this study comparing to those of other metal nanomaterials in previous reports, GQDs comparably improved the device performance^12^,^13^,^14^,^15^,^16^,^17^,^18^,^22^,^23^. Figure 3 and Supplementary Fig. S2 online show the AFM images of GQDs incorporated into PEDOT:PSS films. GQDs were well dispersed in the PEDOT:PSS layer without any significant cracks, due to the high dispersion of GQDs in polar solvents. After the GQDs-incorporated PEDOT:PSS film was formed on the ITO substrate via spin-coating, OPV devices were fabricated following the conventional procedures. Figure 3A,B show AFM topography images of the PEDOT:PSS films w/o and w/GQDs, respectively. In a bare PEDOT:PSS, because it is difficult to form a uniform PEDOT layer on a substrate, the negatively charged PSS is doped in the positively charged PEDOT to enhance the dispersion property in polar solvent. The insulating PSS domains surround the highly conducting PEDOT domains, thereby the grain of PEDOT:PSS is composed of PEDOT-rich core and PSS-rich shell^38^,^39^,^40^,^41^,^42^. In addition, the grain size of PEDOT:PSS is usually determined by hydrogen bonds between PSS-rich shells^43^. Figure 3B indicates that the grain size of GQDs-incorporated PEDOT:PSS film is outstandingly increased while the grain boundaries are spread out more evenly. The GQDs with sufficient oxygen based functionalities intervene in the domain formation between PEDOT and PSS to determine the morphology of PEDOT:PSS film. The negatively charged GQDs can increase the size of PEDOT-rich cores because the positively charged PEDOT polymers combine with GQDs (Supplementary Fig. S3 online). Thereby, the decreased net charge of PEDOT results in lowering the electrostatic interaction between PEDOT-rich and PSS-rich domains. In addition, we obtained Cs-corrected TEM images as shown in Fig. 3C,D in order to understand the positive effects of GQDs in terms of the morphology properties. Unfortunately, it was difficult to visualize their domain structures from the Cs-corrected TEM images because all the components in the PEDOT:PSS layer has similar atoms. However, we observed that the skein-like black wires, which presumably indicated grain boundaries, were untangled in Fig. 3D when compared with Fig. 3C (without GQDs). This results indicate that grain size of PEDOT:PSS film was increased because of the interaction between PEDOT and the incorporated GQDs. The grain size of both PEDOT:PSS films with or without GQDs were quantified using a line measurement with AFM images in Supplementary Fig. S4 online.

### Photovoltaic performance of the OPVs

The GQDs-incorporated device exhibits considerably improved PCE. Here, the PCE was determined by the intensity of power that a device generates using a given power of solar light at the maximum power.


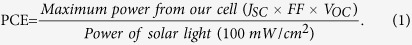


**Figure 4:**
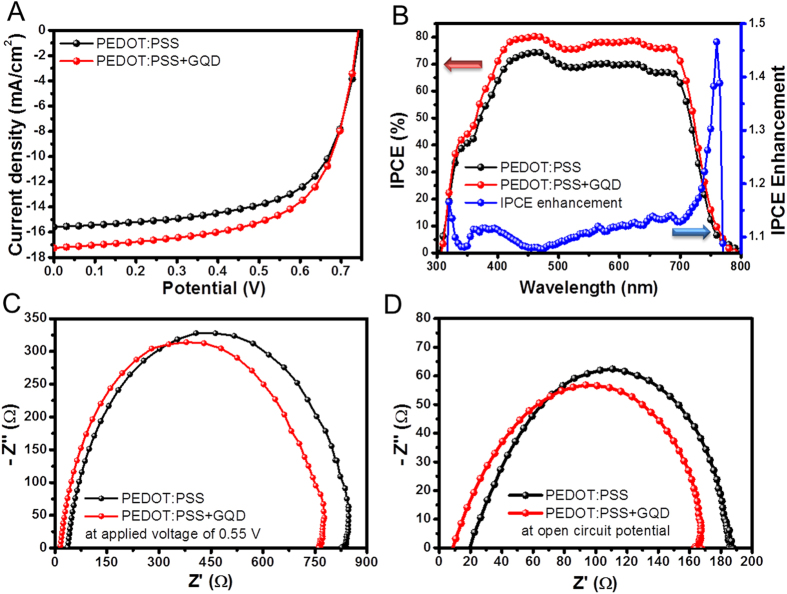
Device performance and characterization. Current vs. potential (J-V) curves (**A**), and incident photon-to-charge-carrier-efficiency (IPCE) and IPCE enhancement factor for the devices (**B**). Nyquist plots of electrochemical impedance spectroscopy at 0.55 V (**C**) and at open circuit potential (**D**). 0.4 wt.% of GQDs was incorporated into PEDOT:PSS layer.

Among the parameters in this study, J_sc_ was significantly improved from 15.6 mA/cm^2^ to 17.3 mA/cm^2^ as shown in Fig. 4A,B, and Table 1. We confirmed that the enhanced J_sc_ was dominantly contributed to the improvement of PCE of the GQDs-incorporated PEDOT:PSS device (GQDs in HTL) through the incident photon to current conversion efficiency (IPCE) measurement. IPCE is defined by the number of injected electrons from the excited cites under the monochromatic light divided by pre-defined input photon flux. Hence, the external quantum efficiency (EQE) can be determined by IPCE measurement. Moreover, the J_sc_ parameter is estimated after integrating all the photocurrent density values obtained from IPCE measurement since the values are plotted as a function of each wavelength^44^.


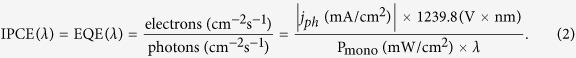


The *j*_*ph*_ and P_mono_ indicate experimentally obtained photocurrent value and power intensity of monochromatic incident light of a particular wavelength λ, respectively.

To understand the enhancement of J_sc,_ the electrochemical impedance spectroscopy (EIS) analysis was carried out as shown in Fig. 4C,D. In the Nyquist plot, the resistance value of the lowest point in the semicircle (near to 0 Ω) is related to the resistance of the both sides of electrodes (anode and cathode) and their interfaces^45^,^46^. In this study, we consider that the reduced resistance value at the lower resistance region resulted from the improved carrier conductance in the ITO/PEDOT:PSS, because there were no changes in the BHJ/cathode part^45^,^46^. The increased PEDOT-rich grains bring about improvement in the current paths and enhancement in the charge conductance^37^,^38^,^39^,^40^,^41^,^42^. Therefore, the hole transport performance of PEDOT:PSS layer with GQDs were improved due to the enhanced efficiency of the charge extraction from the BHJ layer, which resulted in the enhancement of J_sc_ parameter^47^.

Although GQDs absorb visible region in the solar spectrum, the incorporation of GQDs hardly affects the transmittance of PEDOT:PSS layer. Supplementary Fig. S5 B online shows the negligible changes in absorbance of PEDOT:PSS layer between w/o and w/GQDs. The diffuse reflectance data of GQDs/PEDOT:PSS films is slightly enhanced at visible region above 550 nm of solar spectrum (Supplementary Fig. S5 online). In addition, the incorporation of GQDs reduce the overall reflectance of OPV devices (ITO/PEDOT:PSS/BHJ/TiOx/Al) when the incident light is irradiated from the ITO glass side. This reveals that the incident light path from anode to BHJ layer was not disrupted when 0.4 wt.% of GQDs were incorporated into the PEDOT:PSS layer. The IPCE data, in Fig. 4B, shows that the adding of GQDs in the PEDOT:PSS increased the IPCE values in the wavelength range of 300 nm to 800 nm. The IPCE enhancement curve also displays that the increase of wavelength above 550 nm was slightly larger than at shorter wavelength, which results are well matched with the reflectance data in Supplementary Fig. S5 C and D online. This suggests that the improved IPCE result is in reliance with the scattering effect. Unlike the conventional metal-nanoparticle-embedded PEDOT:PSS hole extraction layers, we could neglect the SPR effect on GQDs despite their large amount of electron density. From the calculated E-field intensity distribution of a single Au nanoparticle and GQD, considerable plasmonic effects in the PEDOT:PSS layer with GQDs were not observed (Supplementary Fig. S6 online). Furthermore, the dramatically changed surface morphology of the GQDs-incorporated PEDOT:PSS elongates the incident light path in the device due to the relative index of GQDs (n = 1.6 and k = 0.02 at 600 nm of wavelength)^15^,^16^,^17^,^18^,^48^; this phenomenon is less dominant in the device compared to the morphological tuning effect of the PEDOT:PSS layer.

**Figure 5:**
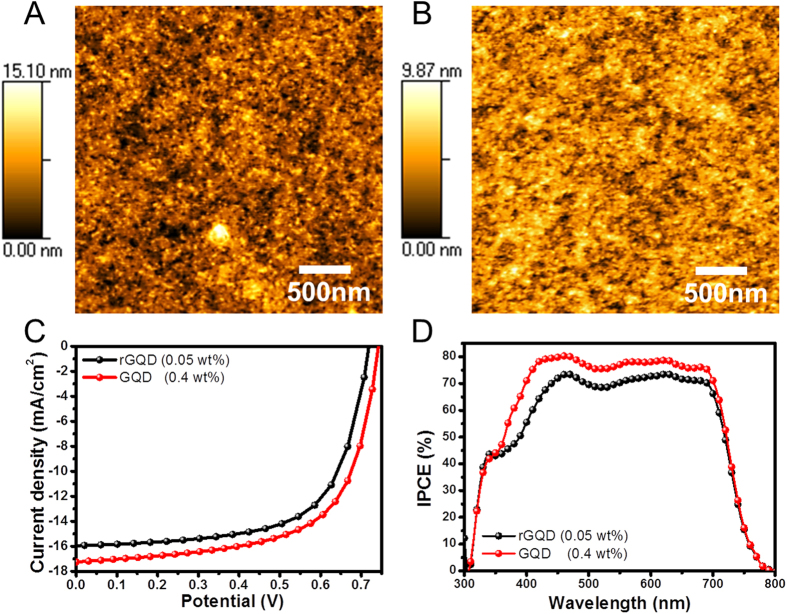
AFM and device performance in comparison with rGQD and GQDs in PEDOT:PSS. AFM images of PEDOT:PSS films incorporating 0.05 wt.% of rGQDs (**A**) and 0.4 wt.% of GQDs (**B**).Current vs. potential (J-V) curves (**C**), and incident photon-to-charge-carrier-efficiency (IPCE) (**D**) for the devices.

Nevertheless the charge conductance and the light harvesting performance were improved with GQDs, the fill factor of GQDs in HTL device was slightly lower than the reference device. This is because of the unconformable energy levels of GQDs as shown in Supplementary Fig. S7 online and slightly low R_sh_ value^49^,^50^ in Table 1. However, this negative effect of GQDs can be overcome by improving the morphological characteristics of composite film, which was confirmed by comparing the dispersion of as-synthesized GQDs and hydrothermally deoxidized rGQDs in the PEDOT:PSS film (Fig. 5)^24^. Previously, it was demonstrated that the oxygen-based moiety signals in XPS or FT-IR were strongly related to the quantitativeness of surface oxygen related functional groups of GQDs. From XPS measurement in Supplementary Fig. S8 online, GQDs contain a much stronger oxygen-based moiety signals than rGQDs. The hydrothermal method caused preponderant sp^2^ carbons in rGQDs so that the oxygen based functionalities of rGQDs were reduced. As shown in AFM images in Fig. 5A,B, even though the amount of rGQDs incorporated into PEDOT:PSS film was 20 times less than GQDs, the aggregated particles were much more observed on the surface of rGQDs-incorporated PEDOT:PSS film. As a result, the device performances might also strongly depend on the film quality of PEDOT:PSS layer as shown in Fig. 5. The rGQD incorporated device optimization information was described in Supplementary Fig. S9 and S10 online.

### Synergistically improved PCE of the OPVs by incorporating GQDs into the polymer layers

**Figure 6:**
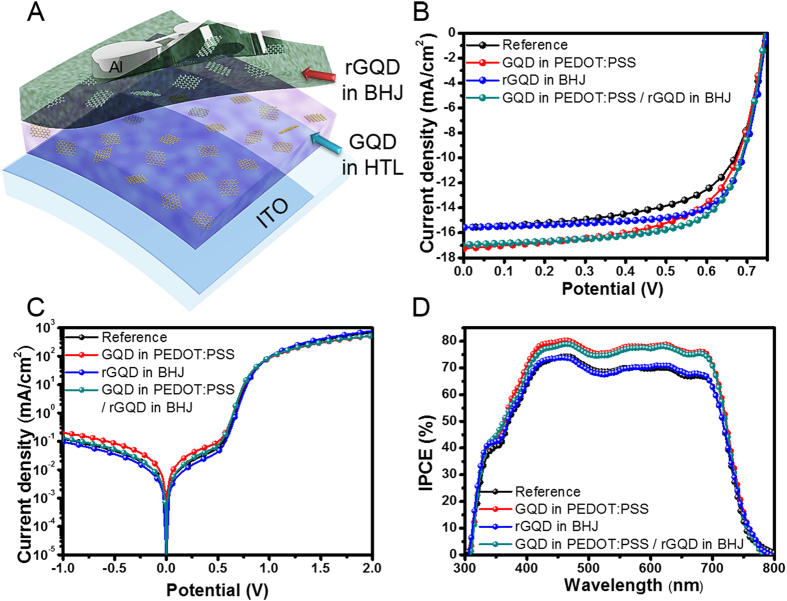
Device performance of rGQDs and GQDs incorporated OPVs. (**A**) Schematic illustration of the device with rGQDs in BHJ layer and GQDs in HTL (PEDOT:PSS), (**B**) J-V curves, (**C**) dark J-V curves and (**D**) IPCE of the devices with plain PEDOT:PSS and BHJ (black), PEDOT:PSS with GQDs (red), BHJ with rGQDs (blue) and GQDs and rGQDs incorporated PEDOT:PSS and BHJ (green). The concentration ratios of GQDs and rGQDs were 0.4 wt.% and 0.02 wt.% respectively.

In our previous results, embedding the rGQDs in BHJ layers have enhanced the device performances of OPVs^24^. To take advantage of the synergistic effects, GQDs and rGQDs were placed in the PEDOT:PSS layer and the BHJ layer, respectively. Figure 6A shows a schematic illustration of the device with the rGQDs-incorporated BHJ film, which was coated on the GQDs-incorporated PEDOT:PSS film. The PCE value of the device composed of the BHJ layer with rGQD and bare PEDOT:PSS (rGQDs in BHJ device) was increased by about 10% as shown in Table 1. The FF was increased from 64.8% for reference device to 71.8% for rGQDs in BHJ device due to the improved carrier conductance and reduced resistance factors. The outstanding synergistic effects were observed in the device with the rGQDs-incorporated BHJ layer and the GQDs-incorporated PEDOT:PSS layer (GQD in PEDOT:PSS/rGQD in BHJ device), representing 8.67% PCE, resulting in 13% increase. The J_sc_ parameter was improved simultaneously with FF parameter. This result reveals that the positive effects from GQDs and rGQDs on the device educe the synergistic enhancement of the OPV performance without impeding each other.

In summary, the hydrophilic GQDs simply derived from carbon fibers were successfully incorporated into the PEDOT:PSS layer of an polymer BHJ photovoltaic device to enhance the power conversion efficiency. The high dispersibility of GQDs in polar solvents such as methanol allowed homogeneous incorporation of GQDs in hydrophilic PEDOT:PSS solution. The incorporation of GQDs with the sufficient oxygen based functionalities led to a significant morphological changes in PEDOT:PSS layer that improved the carrier conductance. Moreover, the GQDs-incorporation induced PEDOT:PSS layer extended light scattering and light confinement inside the OPV device. Taking these advantages of using GQDs in OPVs, the J_sc_ value has been increased by 10%. In addition to the incorporation of GQDs in the hole extraction layer, hydrophobic GQDs that were thermally reduced (rGQD) were hybridized in a bulk-heterojunction layer, which synergistically improved the PCE of OPV devices up to 8.67%.

## Methods

### Synthesis of Graphene Quantum Dots and Reduced Graphene Quantum Dots

GQDs were synthesized by acidic treatments of carbon fiber with 20 ml of HNO_3_ and 60 ml of H_2_SO_4_, and a thermal reaction at 120 °C^36^. After stirred for 24 hours, the mixture was diluted with 800 ml deionized water and neutralized by the addition of Na_2_CO_3_ to obtain a near pH 7. The reduced GQDs (rGQDs) were fabricated by using hydrothermal cutting methods from GOs^24^. The purified GOs, synthesized by the modified Hummer’s method, were treated using thermal reduction at 250 °C for 2 h. The graphite powder was dissolved in an acid solution composed of sulphuric acid and nitric acid to oxidize. After mild sonication for 1 day and dilution in distilled water, the solution was centrifuged for 30 min at 4000 rpm. The rinsing process, dilution and centrifugation, were repeated six times and the rinsed RGO was hydrothermally reduced 200 °C for 10 h to fabricate the rGQDs. After the obtained GQDs and rGQDs solutions were filtered with a 20 nm porous anodisc, it were dialyzed for 3 days to obtain the purified GQDs and rGQDs using a 2000 Da dialysis bag, respectively. Finally, the GQDs were re-dispersed in the methanol solvent for incorporating into the PEDOT:PSS layer. In addition, before preparing the BHJ with rGQDs solution, the rGQDs was re-dispersed in the chlorobenzene solvent.

### Fabrication of OPVs

The hole extraction layer was prepared by incorporating GQDs into PEDOT:PSS (AI4083, Clevious) solution with various weight ratios. The total quantity of methanol used as a co-solvent was same in the all PEDOT:PSS solution. A 40 nm thick PEDOT:PSS layer was spin-cast on a pre-cleaned ITO glass and then dried at 150 °C for 15 min. After that, an 80 nm thick layer of BHJ was spin-coated in an Ar filled glove box. The BHJ solution composed of PTB7 (1-Material Chemscitech Inc., Lot #:YY5220) and PC_71_BM (Nano-c) with 1:1.5 of weight ratio was prepared to 2.5 wt.% in 3% of 1,8-diiodooctane mixed chlorobenzene solvent. In the case of preparatory for devices with rGQDs/BHJ layer, 0.02 wt.% of rGQDs was blended in the BHJ solution. Then, approximately 6 nm of TiOx interlayer was spin-coated for electron conducting layer and a 120 nm thick Aluminum metal cathode was deposited by thermal evaporation at ~10^−7^ Torr.

### Characterization

The AFM images for GQDs were measured by noncontact mode with a Park System XE-100 atomic force microscope. The TEM images were obtained with a JEOL JEM-3010 electron microscope operating at 300 kV and Spherical aberration corrected transmission electron microscopy (Cs-corrected TEM) images were obtained with a JEOL JEM ARM 200 F. In order to prepare the samples for TEM measurement, pre-formed PEDOT:PSS layer was stamping-transferred on a TEM grid by a dry-transfer method with a rigiflex polyurethane acrylate coated polycarbonate (PUA/PC) mold as previously reported method^47^,^51^. The FT-IR spectra were obtained by using a Thermo Scientific Nicolet 6700 spectrometer. XPS analysis was carried out with a Thermo Scientific K-Alpha small-spot X-ray Photoelectron Spectrometer (XPS) system. We prepare our GQD samples for FT-IR and XPS measurements by using the same method with our previous report^24^. The surface topology images of PEDOT:PSS were investigated using atomic force microscopy (AFM, Dimension 3100, Veeco, Plainview, NY) in tapping mode. The absorption and reflectance were obtained using a UV-vis spectrophotometer (UV-3600, Shimadzu). The diffuse reflectance was measured using UV-vis spectrophotometer (Cary 5000) equipped with an integrating sphere accessory. The J-V device performances were measured by a solar simulator (Oriel 91193, 1000 W lamp with 100 mW/cm^2^) using an NREL-calibrated Si solar cell and Keithley 2400 source meters. The incident photon-to-current efficiency (IPCE) measurements were performed by using a Solar Cell QE/IPCE Measurement (Zolix Solar Cell Scan 100).

## Additional Information

**How to cite this article**: Kim, J. K. *et al.* Surface-Engineered Graphene Quantum Dots Incorporated into Polymer Layers for High Performance Organic Photovoltaics. *Sci. Rep.* **5**, 14276; doi: 10.1038/srep14276 (2015).

## Supplementary Material

Supplementary Information

## Figures and Tables

**Table 1:** Performance parameters of the reference device with plain hole transporting layer (HTL, PEDOT:PSS), and the GQDs device composed of GQDs incorporated HTL and rGQD device composed of reduced GQDs incorporated HTL.

	V_oc_ (V)	J_sc_ (mA/cm^2^)	FF (%)	PCE^a^ (%)	R_sh_^b^ (KΩcm^2^)
Reference	0.746 (±0.001)	15.6 (±0.01)	64.8 (±0.13)	7.52 (±0.08)	12.2
GQDs in HTL^c^	0.741 (±0.001)	17.3 (±0.03)	64.0 (±0.16)	8.17 (±0.08)	8.57
rGQDs in HTL^d^	0.719 (±0.012)	15.9 (±0.05)	65.4 (±0.21)	7.48 (±0.14)	18.8
rGQDs in BHJ^e^	0.748 (±0.002)	15.5 (±0.05)	71.8 (±0.20)	8.34 (±0.05)	17.4
GQDs in HTL/rGQDs in BHJ	0.744 (±0.002)	16.9 (±0.08)	68.9 (±0.18)	8.67 (±0.10)	11.5

^a^The device performance was average, as measured by six devices. To determine the cell area, the circular aperture (11.43 mm^2^) was used on top of the active area (15.71 mm^2^).

^b^The shunt resistance values were obtained by using a same calculation process with our previous report^52^.

^c^The 0.4 wt.% of GQDs was incorporated into PEDOT:PSS.

^d^The 0.05 wt.% of rGQDs was incorporated into PEDOT:PSS.

^e^The 0.02 wt.% of rGQDs was incorporated into BHJ.
