# Supplementary material for: Surface-Engineered Graphene Quantum Dots Incorporated into Polymer Layers for High Performance Organic Photovoltaics
Source: Sci Rep. 2015 Sep 22;5:14276. doi: 10.1038/srep14276 (PMC4585740; doi:10.1038/srep14276)
Supplement: Supplementary Information [file srep14276-s1.pdf]

Electronic Supplementary Information

Surface-Engineered Graphene Quantum Dots Incorporated into PEDOT:PSS for High Performance Polymer BHJ Photovoltaics

Jung Kyu Kim,[1,6] Sang Jin Kim,[2] Myung Jin Park,[2] Sukang Bae,[3] Sung-Pyo Cho,[2] Qing Guo Du,[4] Dong Hwan Wang,[5] Jong Hyeok Park,*[6] Byung Hee Hong*[2]

[1] SKKU Advanced Institute of Nanotechnology (SAINT) and School of Chemical Engineering and The Institute of Science and Technology, Sungkyunkwan University, 2066, Seobu-ro, Jangan-gu, Suwon 440-746, Republic of Korea. Fax: +82-31-290-7254; Tel: +82-31-299-4723; E-mail: legkim@gmail.com

[2] Department of Chemistry, College of Natural Sciences, Seoul National University, Daehack-dong, Gwanak-gu, Seoul, 151-747, Republic of Korea. Fax: +82-2-871-6393; Tel: +82-2-880-6559; E-mail: byunghee@snu.ac.kr

[3] Soft Innovative Materials Research Center, Korea Institute of Science and Technology, Eunha-ri san 101, Bongdong-eup, Wanju-gun, Jeollabukdo, 565-905, Republic of Korea.

[4] Institute of High Performance Computing, 1 Fusionopolis Way, #16-16 Connexis North, 138632, Singapore

[5] School of Integrative Engineering, Chung-Ang University, 221 Heukseok-dong, Dongjak-gu, Seoul 156-756, Republic of Korea

[6] Department of Chemical and Biomolecular Engineering, Yonsei University, 50 Yonsei-ro, Seodaemun-gu, Seoul 120-749, Republic of Korea. Fax: +82-2-312-6401, Tel: +82-2-2123-2750; E-mail:lutts@yonsei.ac.kr

*** Address correspondence to** lutts@yonsei.ac.kr (J.H.P.), byunghee@snu.ac.kr (B.H.H)

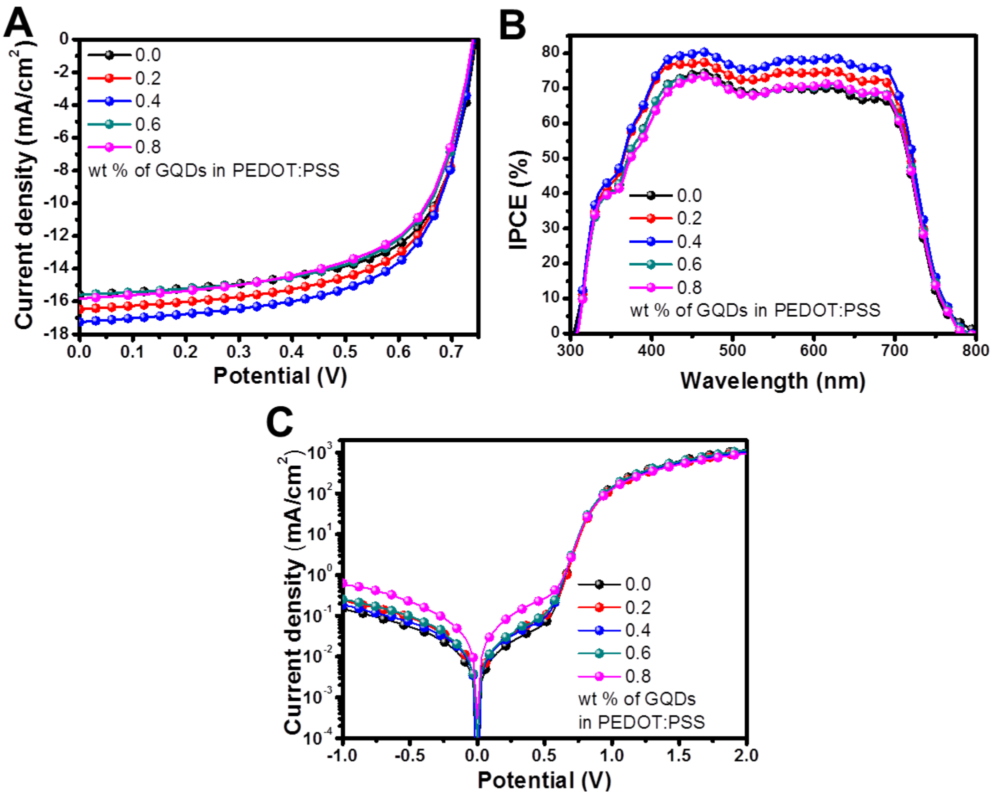

Figure S1. Current vs. potential (J-V) curves (A), incident photon-to-charge-carrier-efficiency (IPCE) (B) and dark current density-potential curves (C) of the devices with various GQD concentrations in the PEDOT:PSS layer. The performance parameters of the devices were exhibited in Table S1.

J_{sc} and FF decreased gradually with further increase of GQDs in PEDOT:PSS. Because GQDs can also absorb over a wide range of the visible spectrum, it was expected that the high contents of GQDs in PEDOT:PSS film was able to decrease the solar light penetration in Figure S5 **A**. In addition, when the concentration of GQDs in the PEDOT:PSS layer exceeded the optimum point, sp^3 carbons on GQDs with functionalities such as hydroxyl, carboxyl and carbonyl groups lead to poor electrical properties, resulting in a decrease of FF value.[2,4]

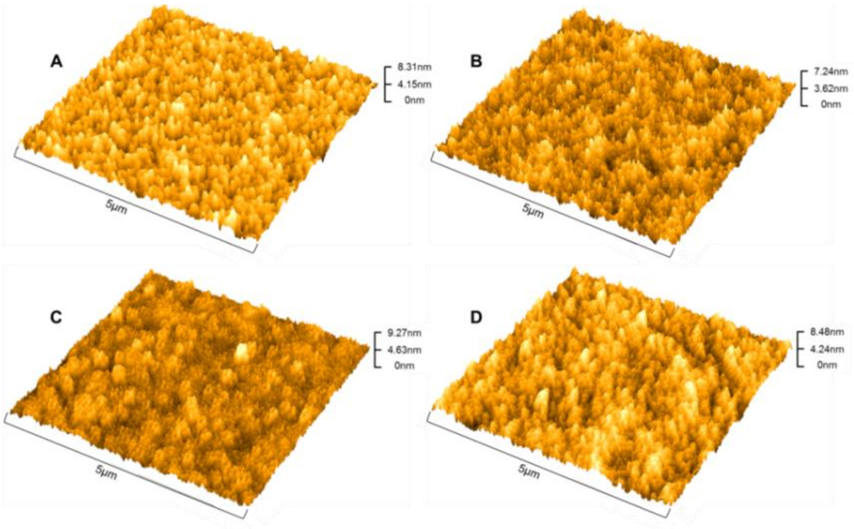

Figure S2. (A) AFM images of the PEDOT:PSS layer without GQDs, and with various ratios of GQDs: (B) 0.2, (C) 0.4 and (D) 0.6.

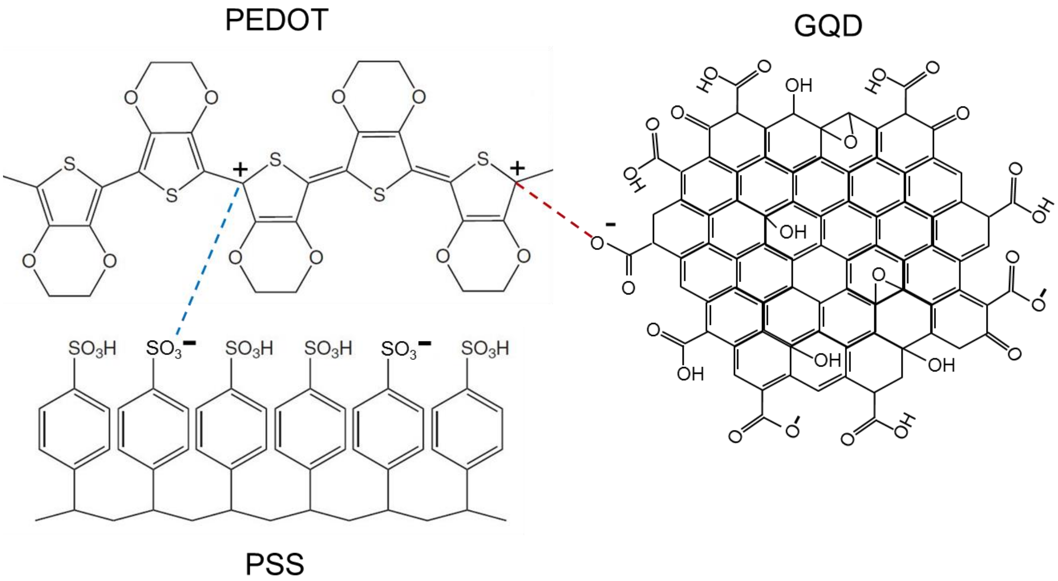

Figure S3. Schematic of chemical structures of PEDOT, PSS and GQD.

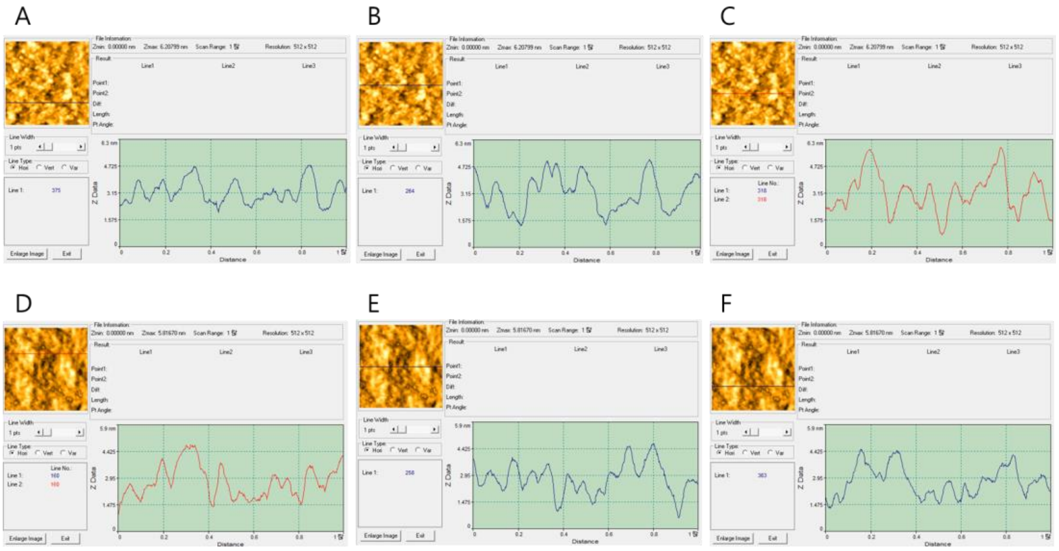

Figure S4. Line measurement results of the surface morphology of the bare PEDOT:PSS film (A)~(C), and the GQD-incorporated PEDOT:PSS film (D)~(F). The average values of the estimated grain size were around 30 nm for the bare PEDOT:PSS, and around 70 nm for the GQD-incorporated PEDOT:PSS. The line measurement was conducted using SPM Lab Analysis software (Veeco instruments, Inc.)

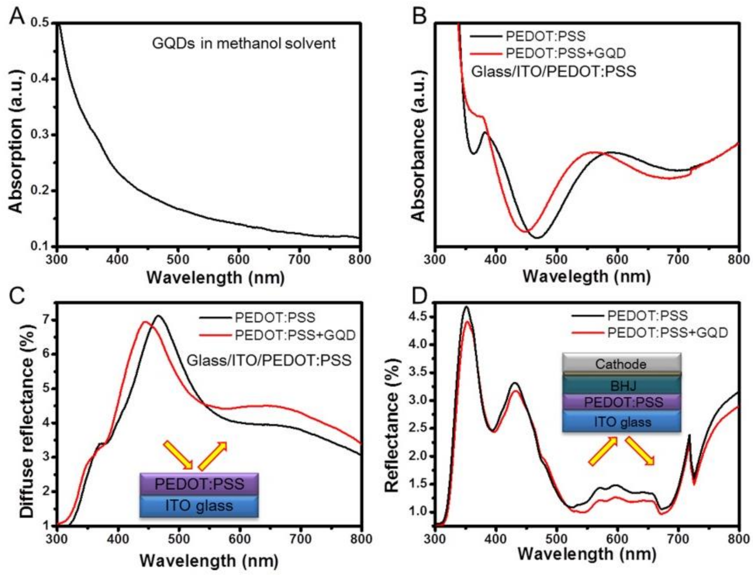

Figure S5. UV- visible adsorption spectra of GQDs in methanol solvent (A). UV-visible adsorption spectra (B) and diffuse reflectance spectra (C) of 40nm-thick PEDOT:PSS films coated on ITO glass. Reflectance spectra of full device structural samples (ITO/PEDOT:PSS/BHJ/TiO_x/Al) where from 700 nm to 750 nm is a lamp changing region (D). 0.4 wt % of GQDs were incorporated into PEDOT:PSS layer.

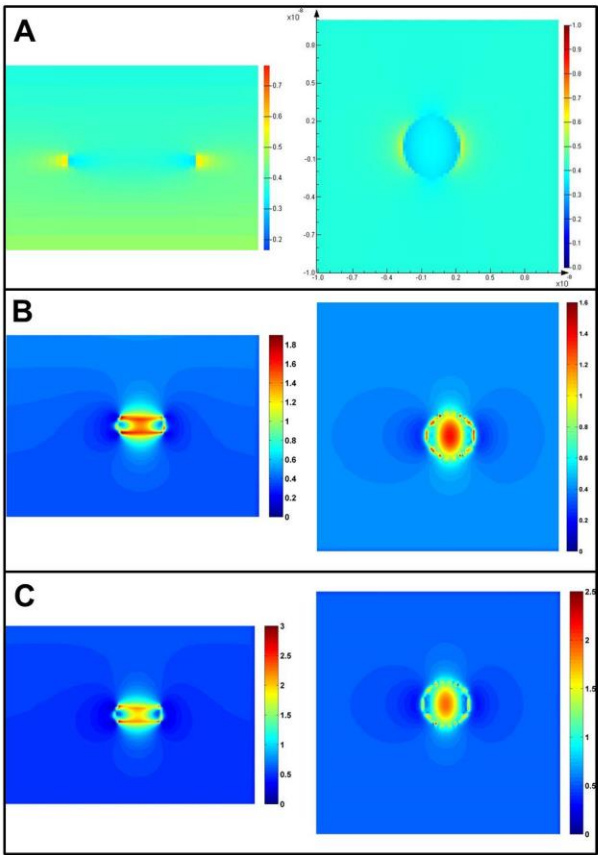

Figure S6. Optical simulation of E-field intensity distribution of GQD obtained by the finite difference time domain (FDTD) method at 500nm (A). (B) and (C) show optical simulation of a gold nanoparticle with the same shape and size as GQDs at 500nm and 600nm respectively.

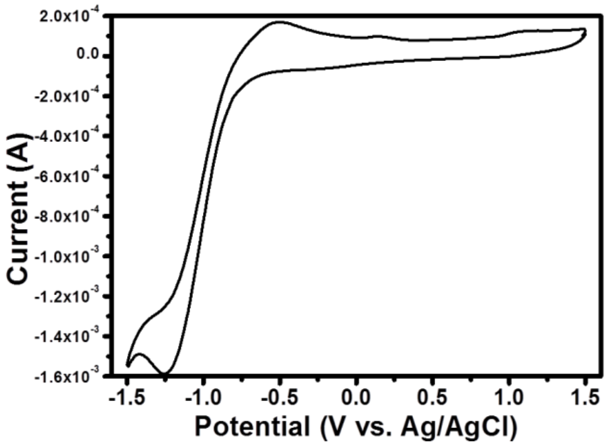

Figure S7. Cyclic voltammetry (CV) curves (scanning rate of 50 mV/s) of GQDs deposited on a platinum (Pt) sheet in acetonitrile solution with 0.1M tetrabutylammonium hexafluorophosphate as an electrolyte. Pt foil and Ag/AgCl were used as the counter and the reference electrodes. GQD solutions were drop-cast on the Pt sheet and fully dried to prepare the working electrode.[5]

While GQDs had -5.5 eV of high HOMO (highest occupied molecular orbital) level[5], PEDOT:PSS had -5.2 eV of work function, so it could result in the formation of leakage current in OPVs, which is a possible reason for the slightly decreased R_{sh}.

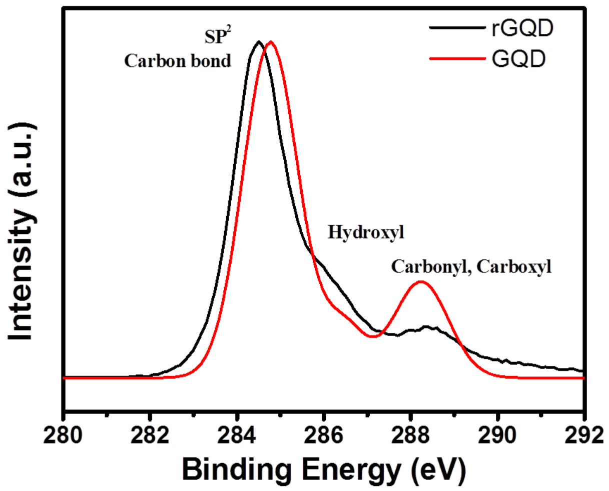

Figure S8. XPS C1s spectra of reduced GQDs(rGQD) and GQDs.

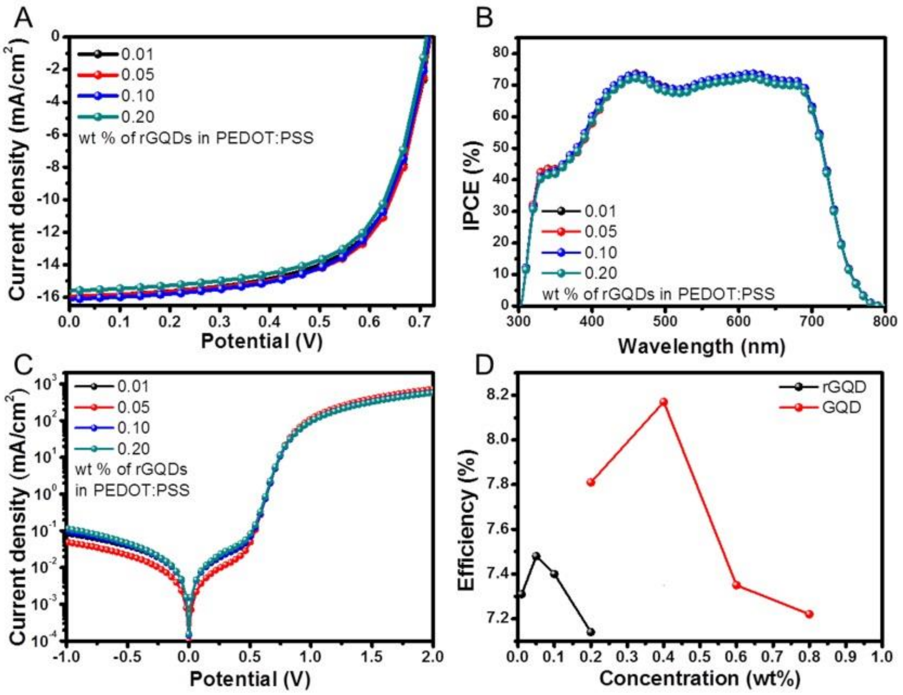

Figure S9. Current vs. potential (J-V) curves (A), incident photon-to-charge-carrier-efficiency (IPCE) (B) and dark current density-potential curves (C) of the devices with various rGQD concentrations in the PEDOT:PSS layer. Efficiency curves of OPV devices according to the GQDs or rGQDs concentration (D), where PEDOT:PSS films were prepared by incorporating rGQDs (black) and GQDs(red). Each point displays the average value of PCE at the concentration.

In our previous report, the resistance through the BHJ solar cell could be improved by incorporating of rGQDs in the BHJ film owing to the high conductivity of sufficient sp^2 carbons in rGQDs, synthesized by the deoxidization process using hydrothermal method. Hence, the shunt resistance value was outstandingly improved.[2] Analogous to that case, the shunt resistance of the device composed of rGQD incorporated PEDOT:PSS layer was much increased as shown in Table 1. However, the poor dispersibility of rGQDs in a polar solvent and PEDOT:PSS solution resulted in mischievous huge aggregations in the PEDOT:PSS film. Thus the contact properties of PEDOT:PSS layer could be deteriorated and this result induced the decrease of V_{oc} value. In addition, aggregated large size of rGQD particles could absorb the light under about 430 nm of wavelength region and quench the light energy into the heat, they might interrupt the light penetration. From this reason, GQDs disturbed the absorption of solar light in BHJ layer, and it resulted in decrease of light harvesting performance as shown in Figure S10. Nevertheless rGQDs had high electrical conductance, the introduction of rGQD-incorporated PEDOT:PSS layer in the BHJ solar cell made a disappointing result in device performance.

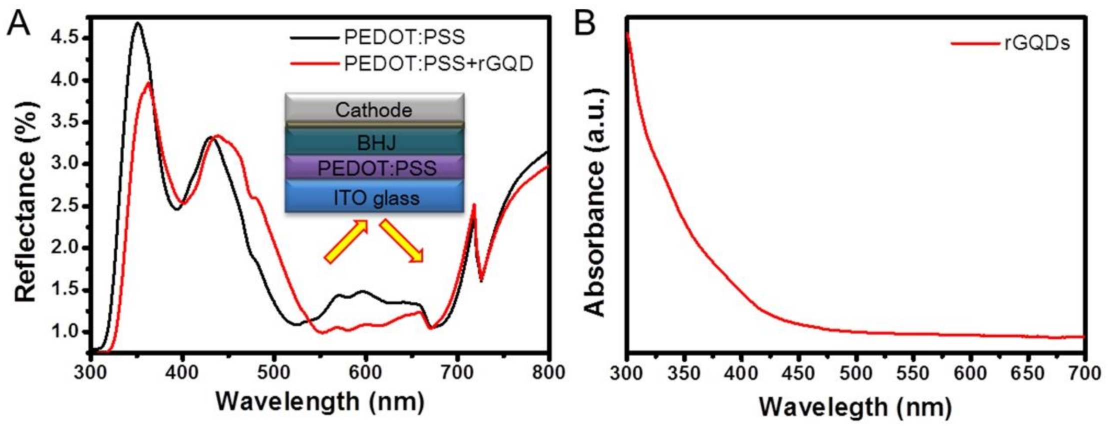

Figure S10. Reflectance spectra of full device structural samples (ITO/PEDOT:PSS/BHJ/TiO_x/Al) where from 700 nm to 750 nm is a lamp changing region, where 0.05 wt % of rGQDs were incorporated into the PEDOT:PSS layer (A). UV- visible adsorption spectra of rGQDs (B).

Table S1. Performance parameters of OPVs with various concentrations of GQDs incorporated into the PEDOT:PSS layer.

GQD (wt %)	V_{oc} (V)	J_{sc}(mA/cm^2)	FF(%)	*PCE(%)	R_{sh}(KΩcm^2)
0.0	0.746 (±0.001)	15.6 (±0.01)	64.8 (±0.13)	7.52 (±0.08)	12.20
0.2	0.740 (±0.001)	16.5 (±0.13)	63.9 (±0.04)	7.81 (±0.07)	8.20
0.4	0.741 (±0.001)	17.3 (±0.03)	64.0 (±0.16)	8.17 (±0.08)	8.57
0.6	0.742 (±0.002)	15.6 (±0.02)	63.6 (±0.23)	7.35 (±0.11)	7.74
0.8	0.740 (±0.001)	15.8 (±0.02)	61.8 (±0.19)	7.22 (±0.08)	3.09

The device performance was average, as measured by four devices. To determine the cell area, the circular aperture (11.43mm^2) was used on top of the active area (15.71 mm^2). The shunt resistance values (R_{sh}) were obtained by using a same calculation process with our previous report.[3]

$$*PCE = \frac{Maximum\ power\ from\ our\ cell\ (Jsc \times Voc \times FF)}{Power\ of\ solar\ light\ (100\frac{mW}{cm^2})}$$

Reference

[1] J. Peng, W. Gao, B. K. Gupta, Z. Liu, R. Romero-Aburto, L. Ge, L. Song, L. B. Alemany, X. Zhan, G. Gao, S. A. Vithayathil, B. A. Kaipparettu, A. A. Marti, T. Hayashi, J.-J. Zhu and P. M. Ajayan, *Nano Lett.* 12 (2012) 844-849.

[2] J. K. Kim, M. J. Park, S. J. Kim, D. H. Wang, S. P. Cho, S. Bae, J. H. Park, B. H. Hong, *ACS Nano*, 7 (2013) 7207-7212.

[3] D. H. Wang, J. K. Kim, J. H. Seo, I. Park, B. H. Hong, J. H. Park, A. J. Heeger, *Angew. Chem.* 125 (2013) 2946-2952.

[4] G. Eda, G. Fanchini and M. Chhowalla, *Nat. Nanotechnol.* 3 (2008) 270.

[5] V. Gupta, N. Chaudhary, R. Srivastava, G. D. Sharma, R. Bhardwaj, S. Chand, *J. Am. Chem. Soc.* 133 (2011) 9960-9963.
